# paraCell: a novel software tool for the interactive analysis and visualization of standard and dual host–parasite single-cell RNA-seq data

**DOI:** 10.1093/nar/gkaf091

**Published:** 2025-02-20

**Authors:** Edward Agboraw, William Haese-Hill, Franziska Hentzschel, Emma Briggs, Dana Aghabi, Anna Heawood, Clare R Harding, Brian Shiels, Kathryn Crouch, Domenico Somma, Thomas D Otto

**Affiliations:** School of Infection & Immunity, University of Glasgow, G12 8TA Glasgow, United Kingdom; School of Infection & Immunity, University of Glasgow, G12 8TA Glasgow, United Kingdom; MVLS SRF, R esearch Software Engineering, University of Glasgow, G12 8QQ Glasgow, United Kingdom; Centre for Infectious Diseases, Heidelberg University Medical Faculty, 69120 Heidelberg, Germany; Institute for Immunology and Infection Research, School of Biological Sciences, University of Edinburgh, EH4 2JP Edinburgh, United Kingdom; School of Infection & Immunity, University of Glasgow, G12 8TA Glasgow, United Kingdom; School of Infection & Immunity, University of Glasgow, G12 8TA Glasgow, United Kingdom; School of Infection & Immunity, University of Glasgow, G12 8TA Glasgow, United Kingdom; School of Biodiversity, One Health & Veterinary Medicine, University of Glasgow, G61 1QH Glasgow, United Kingdom; School of Infection & Immunity, University of Glasgow, G12 8TA Glasgow, United Kingdom; School of Infection & Immunity, University of Glasgow, G12 8TA Glasgow, United Kingdom; School of Infection & Immunity, University of Glasgow, G12 8TA Glasgow, United Kingdom; LPHI, CNRS, INSERM, U niversité de Montpellier, 34090 Montpellier, France

## Abstract

Advances in sequencing technology have led to a dramatic increase in the number of single-cell transcriptomic datasets. In the field of parasitology, these datasets typically describe the gene expression patterns of a given parasite species at the single-cell level under experimental conditions, in specific hosts or tissues, or at different life cycle stages. However, while this wealth of available data represents a significant resource, analysing these datasets often requires expert computational skills, preventing a considerable proportion of the parasitology community from meaningfully integrating existing single-cell data into their work. Here, we present paraCell, a novel software tool that allows the user to visualize and analyse pre-loaded single-cell data without requiring any programming ability. The source code is free to allow remote installation. On our web server, we demonstrated how to visualize and re-analyse published *Plasmodium* and *Trypanosoma* datasets. We have also generated *Toxoplasma–*mouse and *Theileria–*cow scRNA-seq datasets to highlight the functionality of paraCell for pathogen–host interaction. The analysis of the data highlights the impact of the host interferon-γ response and gene expression profiles associated with disease susceptibility by these intracellular parasites, respectively.

## Graphical Abstract

**Graphical Abstract:**
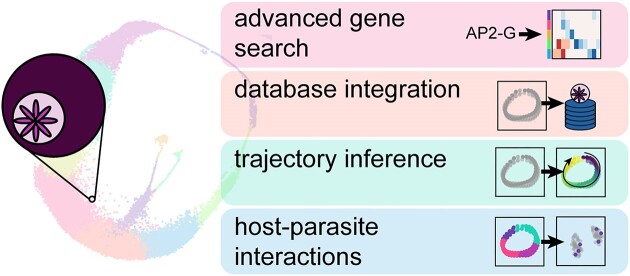

## Background

Single-cell transcriptomics sequencing (scRNA-seq) was first introduced in 2009 [1] and has since had a dramatic impact on the investigation of cellular heterogeneity, the identification of novel cell types, and the discovery of new drug targets [2]. It has also enabled projects such as the Human Cell Atlas, which seeks to catalogue and describe every cell type in the human body [3].

Single-cell sequencing has enabled similar cell ‘atlas’ projects in the field of parasitology, typically detailing the full life cycle of various parasite species with complex life cycles encompassing a range of ecological niches and morphologies such as *Plasmodium* [4], *Toxoplasma* [5], *Cryptosporidium* [6], or *Schistosoma* [7]. Other applications include the in-depth description of specific life stages [8] or sexual development [9] in parasites. Additionally, the investigation of host–parasite interactions (HPIs) can reveal differential host immune responses and parasite invasion preferences for different cell types [10].

HPI analysis is enabled by dual RNA-seq, a novel transcriptomic approach [10] for capturing gene expression profiles linked to cellular infection. Dual scRNA-seq can be used to capture both host and parasite messenger RNA (mRNA) transcripts from infected cells, enabling the investigation of HPIs on the single-cell level and allowing the complementary interrogation of critical processes such as infection, pathogenesis, and cellular immunity. This technique is particularly useful in the study of intracellular parasites: in *Theileria*, which is known to induce an oncogenic state in infected bovine leukocytes [11]; in *Toxoplasma*, the cause of fatal toxoplasmosis [12], which secretes hundreds of proteins to alter the infected host cell transcriptome and phenotype; and finally, in *Plasmodium*, the causative agent of malaria [9]. However, there is a marked lack of dedicated tools for analysing host–parasite dual scRNA-seq datasets [13].

Single-cell datasets are inherently collaborative projects, relying on effective cell culturing or sample collection by biologists in the wet lab and statistical analysis by bioinformaticians *in silico*. Analysis of single-cell transcriptomic data is typically handled by general-purpose packages such as Seurat [14] or Scanpy [15], often in combination with more specific tools and/or software packages designed to customize specific steps in the analysis pipeline, such as cluster annotation, differential gene expression analysis, or trajectory inference [16].

However, computational analysis is most often the sole responsibility of a trained bioinformatician, with other, wet lab-based members of the collaboration having relatively restricted access to this process. This can be a limiting factor in research projects, as in-depth biological knowledge that wet lab-based collaborators possess can provide key insights needed to guide the further hypothesis-driven analysis of single-cell datasets and formulate experiments for validation of bioinformatic prediction.

Cell atlases (such as the Human Cell Atlas, the Malaria Cell Atlas [4], the cell atlases made available via VEuPathDB [17], and the SchistoCyte Atlas [18]) help the field of single-cell transcriptomics overcome analysis bottlenecks, facilitating effective collaboration between biologists and bioinformaticians by visualizing single-cell datasets via single-cell browsers. These browsers are software tools that automate the visualization of single-cell data, providing users with interactive graphical interfaces that remove the requirement for computational skills needed to meaningfully interact with scRNA-seq data. These platforms address a common problem inherent to the multi-disciplinary nature of many current biomedical research projects—a disconnect between the experimental scientists who generate material for and validate data predictions and the computational scientists who generate the bioinformatic data [19]—by providing a single, shared representation of a given dataset around which to base discussions.

Popular single-cell browsers include the UCSC Cell Browser [20], the Single Cell Explorer [21], and CELLxGENE [22], a Chan Zuckerberg Initiative project that was recently identified as the premiere option for the publication of single-cell data [23] and that is used to visualize datasets hosted on the Human Cell Atlas. CELLxGENE is distinguished not only by its fully comprehensive user interface and efficient implementation (which allows for the visualization of millions of cells [22]), but also by its inclusion of in-built options such as differential expressed gene (DEG) analysis and marker gene identification, a notable departure from other single-cell browsers, which typically focus exclusively on data visualization (Table 1). CELLxGENE is used in VEuPathDB [17] to host single-cell datasets derived from parasites and their hosts. However, the focus of CELLxGENE leans more towards data presentation rather than analysis.

**Table 1.** Comparison of paraCell to other available single-cell browsers [23]

	UCSC Cell Browser	Single Cell Explorer	iSEE	CELLxGENE	CELLxGENE VIP	paraCell
Web sharing	✓	✓	✓	✓	✓	✓
Interactivity	✓	✓	✓	✓	✓	✓
Cell select functionality	✓	✓	✓	✓	✓	✓
Multiple embeddings		✓	✓	✓	✓	✓
DEG analysis			✓	✓	✓	✓
Additional visualization options					✓	✓
Additional search options						✓
External database links						✓
Trajectory inference						✓
Host–parasite interaction analysis						✓
Docker				✓		✓

The third-party plugin CELLxGENE VIP seeks to overcome this limitation by providing users with additional options for the analysis and visualization of single-cell data [24], such as heatmaps, violin plots, and volcano plots. However, while VIP significantly expands the number of analysis options available within the CELLxGENE framework, it still has limitations. VIP does not provide users with the ability to search a given dataset using gene product names or gene ontology (GO) terms and does not include any provision for trajectory inference, enrichment analysis after differential expression, or the interrogation of the dual scRNA-seq datasets required for study of HPI, functionalities that would be highly appreciated in the field of parasitology. Thus, CELLxGENE VIP is very similar to the other actively maintained cell atlas services, none of which provide all these options (Table 1).

Here, we present paraCell, a novel software tool based on CELLxGENE and VIP, designed for the visualization and analysis of single-cell parasitological data. Example paraCell atlases are available at http://cellatlas.mvls.gla.ac.uk. Five use cases are detailed in this paper, including two novel dual scRNA-seq host–parasite datasets describing the impact of *Theileria* infection on either *Bos indicine* or *Bos taurine* cattle and *Toxoplasma*-infected bone marrow-derived macrophages treated with interferon-γ (IFN-γ).

## Materials and methods

### Software architecture

paraCell makes minimal changes to the server–client architecture established by CELLxGENE VIP, which combines an interactive front end built via JavaScript libraries such as D3 and jQuery with a Flask back end that implements popular scRNA-seq analysis platforms such as Seurat (v3.2.3) and Scanpy (v1.6.1) on the server side [24]. All this functionality is presented as a plugin built on top of the base CELLxGENE interface.

Instead, paraCell utilizes the modular nature of CELLxGENE VIP to extend the plugin, incorporating additional resources on the front end (e.g. Fuse.js) as well as the back end (e.g. tradeSeq).

paraCell is also available as a containerized application, minimizing set-up requirements for the user. To install paraCell on a stand-alone server, a macOS or Debian GNU/Linux machine with at least four CPU cores and 8 GB RAM is recommended.

### Input data format

The only input paraCell requires is a properly formatted scRNA-seq AnnData file (.h5ad file extension).

The AnnData file format, initially developed by Scanpy, is optimized for the storage and manipulation of annotated matrices and has become a popular standard file format within single-cell genomics. In the context of scRNA-seq, AnnData files combine a central cell-by-gene count matrix (*X*) with slots holding cell annotations (obsm, obsp), gene annotations (var), and unstructured annotations (uns) into a single data structure.

CELLxGENE requires input AnnData files that fulfil two criteria—cell barcodes and/or gene names must be unique, and at least one embedding must be available in the obsm slot.

CELLxGENE VIP incorporates an optional text file as input, letting users display additional information in an atlas (e.g. a description of the dataset) and set initial visualization options [24].

paraCell simplifies the user experience and takes full advantage of the AnnData format by storing all the information necessary to run the plugin in ‘paraCell_setup’—a Python dictionary stored in the uns slot. This dictionary not only fully replaces the text file used by CELLxGENE VIP but also controls the activation of core paraCell features. Only features relevant to the dataset in question are activated during set-up, preventing user confusion.

Users are provided with a script capable of inserting the ‘paraCell_setup’ dictionary into a targeted AnnData .h5ad file—further guidance on the use of ‘paraCell_setup’ is available on the paraCell ‘Wiki’ at (https://github.com/sii-cell-atlas/paraCell/wiki/Setting-Up-paraCell).

It should be noted that the primary analysis of processing raw data and quality control, including the above instruction to load the file into the paraCell framework, is best performed by a bioinformatician.

### External database links

paraCell links cell atlases to external databases: currently available options are NCBI and VEuPathDB. The specific database linked to an atlas is established within the ‘paraCell_setup’ dictionary.

Multiple paraCell tabs return results in the form of data tables. Any gene names included in such tables are automatically reformatted into hyperlinks, pointing towards the relevant gene profile page on the pre-specified external database system.

### paraCell tabs

The analysis, visualization, and utility options provided by paraCell are presented as additional tabs within the original CELLxGENE VIP plugin window. paraCell also makes changes to the ‘Add Genes’ tab provided by CELLxGENE VIP, extending its functionality to incorporate links to external database systems and base CELLxGENE gene sets.

### Gene set import

Gene sets created using base CELLxGENE functionality are automatically made available for import by paraCell increasing interoperability between the plugin, and core framework.

Available gene sets can be selected and imported via a dropdown menu in the ‘Add Genes’ tab.

In addition, the Search Database with User Defined Gene Set paraCell option made available in the ‘Add Genes’ tab uses available gene sets as search strategies on a pre-specified database system.

### Help

The ‘Help’ tab provides a basic overview of paraCell and its functionality, as well as links to CELLxGENE VIP and the paraCell Wiki, which provides comprehensive tutorials on the functionality of each tab.

The paraCell Wiki can be found at https://github.com/sii-cell-atlas/paraCell/wiki.

Each individual paraCell tab also includes a link to a corresponding tutorial page on the paraCell Wiki.

### Downloading the result files

Beside the graphical interface with different plotting functionality, it is possible to download all results tables produced by paraCell as CSV files.

### Advanced gene search

The ‘Advanced Gene Search’ tab includes two additional search bars, expanding the range of data types that can be used to search a paraCell atlas to include gene names, gene functions, and GO terms. Look-up tables for each option are stored within the AnnData object, which relates the data type to the gene ID indices used by CELLxGENE.

These look-up tables are automatically parsed upon page launch, and the contents of each stored as ordered vectors on the front end, enabling the quick retrieval of results upon user input. Results are retrieved automatically via a fuzzy search method enabled by Fuse.js.

### Cell population view

The ‘Cell Population View’ tab utilizes Plotly.js to create interactive scatter graphs of gene expression within a single cell population, split between two conditions. A user-specified gene-level annotation in the AnnData object can then be added to the graph as hover data.

Results are also available as a jQuery DataTable of differentially expressed genes, obtained by applying the diffxpy (https://diffxpy.readthedocs.io/, version 0.7.4) Welch’s *t*-test algorithm. The data are filtered to only contain cells belonging to the two conditions being compared prior to the DEG analysis.

### Trajectory inference

The ‘Trajectory Inference’ tab lets users present pre-computed Slingshot lineages within paraCell. The coordinates for each lineage are stored as tables in the underlying AnnData object. The embedding graph used to generate the lineages is indicated via the ‘pseudoEmbed’ key of ‘paraCell_setup’ and is coloured according to the levels of a user-specified categorical annotation.

Novel trajectory inference results can also be generated via the Scanpy implementation of the partition-based graph abstraction (PAGA) algorithm, based on a user-specified embedding graph and categorical annotation.

Users are provided with example scripts detailing the preparation of an AnnData object for the Trajectory Inference tab in the ‘slingshot_example’ directory provided in the GitHub repository.

Further guidance on the Trajectory Inference tab, as well as a link to the official Slingshot tutorial, is available in the paraCell Wiki.

### tradeSeq

The ‘TradeSeq’ tab enables the presentation of results generated via tradeSeq (v1.6.0) within paraCell. The tradeSeq R package analyses differential gene expression along trajectories in SingleCellExperiment (SCE) [25] objects.

tradeSeq uses a general additive model (GAM) to estimate smooth functions (smoothers) of gene expression along the pseudotime variable of each lineage. tradeSeq is compatible with a wide variety of dimension reduction and trajectory inference methods and is applicable to both simple and complex trajectories [26]. Rpy2 (v3.3.5) allows R scripts to run embedded in a Python process, enabling the paraCell back end to interact with tradeSeq.

tradeSeq includes several tests capable of identifying differential gene expression patterns both within and between lineages. paraCell can present pre-computed results generated via associationTest(), a tradeSeq package function that assesses whether gene expression is associated with pseudotime either globally or for each specific lineage.

Test results are extracted from the SCE object that tradeSeq operates on, stored as a table in the AnnData object, and then presented as a jQuery DataTable within the TradeSeq tab.

paraCell can also plot the smoothers estimated by tradeSeq, visualizing the relationship between a given gene and the progression of specified lineages. This function requires that results of the GAM fitting process are extracted from the SCE data object, split into columns, and saved into the AnnData object as a collection of vectors.

paraCell provides users with scripts that automate both the extraction of tradeSeq results from a given SCE data object and the addition of those results to a user-specified AnnData object, streamlining the use of the TradeSeq tab.

Further guidance on the TradeSeq tab, as well as a link to the official tradeSeq tutorial, is available in the paraCell Wiki.

### Host–parasite interactions

The ‘Host–Parasite Interactions’ tab is designed for the analysis of dual scRNA-seq datasets in which each cell contains both host and parasite genes.

The Host–Parasite Violins option generates a multi-panel violin plot indicating the percent concentration of host or parasite genes within each level of a specified categorical annotation. This requires that the percent host and parasite content for each cell is pre-calculated and stored as a cell-level annotation in the AnnData object.

Host and parasite uniform manifold approximation and projections (UMAPs) are generated by subsetting the AnnData object to contain exclusively host or parasite genes, before reducing dimensions and clustering via Scanpy. Host/parasite subsetting relies on pre-made vectors listing every host and parasite gene in the dataset, respectively (host_genes, parasite_genes), stored in the AnnData object.

Both UMAPs are presented as interactive scatter graphs via Plotly.js. Cell selections made via the Plotly.js lasso select function on one graph (e.g. Host UMAP) automatically apply one of two update options to the opposing graph (e.g. Parasite UMAP). The ‘Highlight Selected Cells’ update option alters the traces on the opposing graph, colouring cells with the same cell IDs as the selected cells red and all other cells blue. The ‘Recluster’ update option regenerates the opposing graph, this time subsetting the data object to only contain the selected cells in addition to the original host/parasite gene subset.

Host/parasite specific gene markers are generated for the levels of specified categorical annotation by subsetting the AnnData object to contain only host or parasite genes, before DEG analysis via the *t*-test, *t*-test with overestimated variation, or Wilcoxon Rank Sum options provided by Scanpy.

An example use case demonstrating the generation and preparation of a host–parasite AnnData object for paraCell can be found in the ‘host_parasite_example’ directory provided in the GitHub repository.

### Generation of figures

To generate high-quality figures in paraCell, we changed the parameters in the ‘Global Setting’ tab (from CELLxGENE VIP) and set the ‘Figure format’ to SVG or PDF, being sure to regenerate any plots after applying this change. PNG can also be generated to 600 DPI. For the figures in this paper, we used these methods; however, some figures were also edited using Adobe Photoshop.

### Parameters for case studies 2 and 3

Default parameters in paraCell were used.

### Data generation

#### *Toxoplasma gondii* tachyzoite cell culture

Wildtype (WT) *T. gondii* tachyzoites were grown at 37°C with 5% CO_2_ in human foreskin fibroblasts cultured in Dulbecco’s modified Eagle’s medium (Thermo Fisher Scientific) supplemented with 3% Fetal Bovine Serum, 2 mM l-glutamine, and penicillin–streptomycin.

#### Mice

C57BL/6 (B6) mice (JAX 000664) purchased from Jackson Laboratories. All mice were female, aged between 6 and 12 weeks. Procedures were conducted in accredited animal facilities under the project license PPL30/3423, authorized by the UK Home Office Animal Procedures Committee.

#### BMDM isolation and infection with T*. gondii*

Six- to twelve-week-old B6 mice were euthanized and femur and tibia bones were pooled from two to three mice. Tissue was removed from the bones using forceps, bones were placed in ethanol for 30 s to sterilize, and then transferred to 1× sterile Phosphate-Buffered Saline (PBS). Bone marrow was extracted and resuspended in 1 ml ACK lysis buffer (Thermo Fisher Scientific). Cells were then incubated in ACK lysis buffer for 2–5 min before washing. Bone marrow-derived macrophages (BMDMs) were cultured in RPMI (Thermo Fisher Scientific) complete media, supplemented with 10% filtered FBS, 2 mM of l-glutamine, penicillin–streptomycin, and 50 μM β-mercaptoethanol (Thermo Fisher Scientific). Cells were diluted to 5 × 10^5^ cells/ml and 30 ng/ml of M-CSF (BioLegend) was added to promote BMDM differentiation. Culture medium was replaced with fresh complete Roswell Park Memorial Institute (RPMI) and 30 ng/ml M-CSF 3–4 days following isolation.

After 6 days of differentiation, BMDMs were infected with WT RHΔKu80 *T. gondii* tachyzoites at an Multiplicity of Infection (MOI) of 1.5, in the presence or absence of 20 ng/ml murine recombinant IFN-γ (BioLegend). Cells were incubated at 37°C with 5% CO_2_ for 24 h before harvesting for scRNA sequencing.

#### BMDM sample preparation for 10× Genomics scRNA sequencing

Briefly, cells were washed twice with sterile PBS and then incubated with 4 ml of trypsin at 37°C with 5% CO_2_ for 5–7 min. Six millilitres of complete RPMI medium was then added to gently resuspended the cells. Cells were spun down at 1500 × *g* for 5 min, resuspended in 10 ml complete RPMI medium, and then filtered using a 40 μM filter before diluting in 0.2% trypan blue and counting. For 10× scRNA sequencing analysis, 1.6 × 10^4^ cells/condition were resuspended in 0.04% Bovine Serum Albumin (BSA) in PBS. Cells were counted again and loaded onto the 10× Genomics technology for single-cell RNA sequencing.

#### Single cell preprocessing

The raw fastq files were mapped with Cellranger (version 7.0.0) against a combined reference of *T. gondii* (version 59 VEuPathDB) and mouse (version GRCH38_mm10_ensemble93). With custom scripts, we extended the parasite’s UTR by 2.5 kb. The reason for extending the UTR is that 10× Chromium is a 3′UTR enriched method, and many annotations do not have those accurately annotated, as explained in [27]. If the extended UTR reached another genome feature, it was shortened accordingly to ensure there was no overlap. The read count matrix was analysed with Seurat (version 3) following the integration tutorial. We chose the following parameters: cells must have between 200 and 5000 expressed genes, default Seurat integration, 30 principal component analysis (PCA) dimensions, and a resolution of 0.5. The resulting object was saved as an AnnData .h5ad file for paraCell.

#### Cow–Theileria

Two *Theileria* infected cell lines were utilized. One line [Sah1 (82H)] represented an *ex vivo* isolate derived from an infected calf of the Sahiwal breed (*Bos indicus*), while the other [Hol 3 (12 886)] was derived from an infected Holstein calf (*Bos taurus*), as reported in [28]. Both Sahiwal and Holstein animals were infected with sporozoites from the same *T*. *annulata* Hissar stock and cell lines cultured as standard in RPMI 1640 medium (Sigma–Aldrich) supplemented with 10% FCS, 4 mM l-glutamine, and 50 μM β-mercaptoethanol, at 37°C for a limited period (l2 passages). Both cell lines were fully established based on the level of infection (>95% macroschizont infected cells). Cultures were set up at 1 × 10^5^cells/ml and cultured for 48 h. Cell counts and viability were determined by trypan blue exclusion using a Countess™ 3FL automated cell counter (Invitrogen). Cell viability was 94% (Sah 1) and 95% (Hol 3). Cell growth for the Holstein line was 1.58-fold greater than the Sahiwal.

Multiome nuclei extraction protocol was adapted from (https://doi.org/10.17504/protocols.io.rm7vzyx75lx1/v1). Cells were harvested by centrifugation (400 × *g* for 10 min) and lysed for 10 min on the rotor at 4°C with 1× CST lysis buffer (as indicated in https://doi.org/10.17504/protocols.io.rm7vzyx75lx1/v1). Lysate was filtered with a 40 μm mini cell strainer into a clean pre-labelled 5.0 ml Eppendorf tube and PBS + 1% BSA added to dilute the lysis buffer. The tube was spun 500 × *g*, 4°C for 5 min. Nuclei were resuspended and counted with a Neubauer haemocytometer; 10 000 nuclei were loaded for each chip line on a 10× Genomics Multiome J chip (#1000230). (We needed to use the nuclei in order to try the multiome kit, a combination of scRNA-seq and scATAQ-seq. However, the scATAQ-seq failed.) The RNA gene expression libraries were prepared according to the 10× Genomics Multiome protocol (#1000285, #1000215, #1000212) and sequenced at the Glasgow University Polyomics facility on an Illumina NextSeq 2000.

#### Single-cell pre-processing

The raw fastq files were mapped with Cellranger (version 7.0.0) against a combined reference of *T. annulata*(Ankara) (version 59 VEuPathDB) and *Bos taurus* genome from Ensembl (version CowARS-UCD1.2_UTR). With custom scripts, we extended the UTR of the parasite by 2.5 kb. The read count matrix was analysed with Seurat (version 3) following the integration tutorial. We chose the following parameters: host cells must have between 1000 and 7500 expressed genes and maximal 2500 (Sahiwal) and 5000 (Holstein) parasite UMIs. As we were interested in HPI, we limited the 25% of parasite transcripts per cell. For the integration, we used default Seurat (Version 4), 30 PCA dimensions, and a resolution of 0.5. The resulting object was saved as an AnnData .h5ad file for paraCell.

The paraCell marker function ‘Marker Genes’ tab was used to identify markers for the seurat_clusters with default parameters. These markers were then loaded with the ‘Add Genes’ tab and visualized using paraCell’s ‘Heatmap’ tab (derived from CELLxGENE VIP). Differentially expressed genes were identified using the ‘DEG’ tab, applying Welch’s test (CELLxGENE), FDR < 0.05, and absolute log_2_FC > 0.6; gene set enrichment analysis (GSEA) was performed using the C2.all.v7.2 database with default parameters. The figure displays selected GO terms from the top 10 upregulated and top 10 downregulated pathways.

## Results

We implemented paraCell as an expansion of CELLxGENE and VIP by adding new analysis, visualization, and search options to the application. We also increased interoperability between the plugin and base CELLxGENE, as well as external database systems such as NCBI [29] and VEuPathDB.

paraCell and its dependencies can be installed with Anaconda, using an environment file contained within the source code. Once installed, paraCell facilitates the transformation of AnnData objects [15] into either local or online cell atlases (see the ‘Materials and methods’ section).

Like CELLxGENE and VIP, paraCell depends on the AnnData object—a standard and highly flexible file format capable of holding all the diverse information associated with a given scRNA-seq experiment, from gene expression counts to cell annotations. This adaptability allows the AnnData file to include unstructured metadata, in the form of a Python dictionary labelled ‘paraCell_setup’, that is necessary to parametrize paraCell. Further information on the creation and use of ‘paraCell_setup’ can be found in the ‘Wiki’ section of the paraCell GitHub repository (https://github.com/sii-cell-atlas/paraCell). Multiple running instances of paraCell can be accessed via http://cellatlas.mvls.gla.ac.uk/, including the five case studies described below.

### Comparison to other available tools

CELLxGENE VIP extends the CELLxGENE framework, and paraCell builds on CELLxGENE VIP, resulting in a platform distinguished by an intuitive user interface and a comprehensive suite of additional analysis and visualization options (Table 1). paraCell benefits from active development in the CELLxGENE community and is further differentiated from the alternatives by its focus on parasitology, providing functionality such as links to the popular parasite data warehouse VEuPathDB, additional annotation options in DEG analysis, and provisions for HPI analysis.

The following five sections demonstrate the unique capabilities of paraCell using case studies based on parasite scRNA-seq datasets. The paraCell workflow associated with each case study can be found on the paraCell GitHub Wiki.

### Case study 1: advanced search options

A common first step taken by parasitologists when interacting with a single-cell dataset is to check whether any cells express a specific gene of interest. Although base CELLxGENE does include a search bar, this native search functionality is limited to a single identifier, typically the gene ID or gene name. paraCell seeks to overcome this limitation by implementing advanced search options. Here we demonstrate the utility of these options in a paraCell atlas describing scRNA-seq *Plasmodium berghei* data generated by Hentzschel *et al.* [9].

paraCell implements an additional search bar capable of searching both colloquial gene names and gene product descriptions as identifiers. In-built autocomplete functionality also enables the use of gene ‘terms’ as identifiers. For example, we used the term AP2, which is an important transcription factor family in *Plasmodium*, and other clinically relevant apicomplexans, to identify all AP2-related genes in the dataset. Users can then save specific items retrieved via this search bar to a custom gene set, enabling the generation of a variety of informative plots via CELLxGENE VIP-derived functionality (e.g. violin plots depicting the expression of each gene within specific groupings of cells). A walkthrough tutorial and the results of this example are available on the paraCell GitHub Wiki (https://github.com/sii-cell-atlas/paraCell/wiki/1.-Advanced-Gene-Search).

paraCell also implements a GO search bar, capable of retrieving all genes associated with a specific GO term and saving the retrieved items as a gene set for later use with CELLxGENE VIP-derived analysis and/or plotting functions.

### Case study 2: DEG and integration of external databases

CELLxGENE includes native DEG analysis functionality, which lets users select and then compare two specific cell populations at the transcriptomic level. The top genes showing elevated expression in each population are saved and presented to the user as gene sets.

paraCell enables users to import these gene sets directly into the plugin. Any gene set available in paraCell can then form a search strategy on a preset external database: currently available options are VEuPathDB or the NCBI gene database. The database associated with a given cell atlas is extracted from metadata stored in the underlying AnnData object.

This approach empowers users to combine the functionality of CELLxGENE and paraCell with that of external databases, such as the GO analysis option built into VEuPathDB.

**Figure 1.**
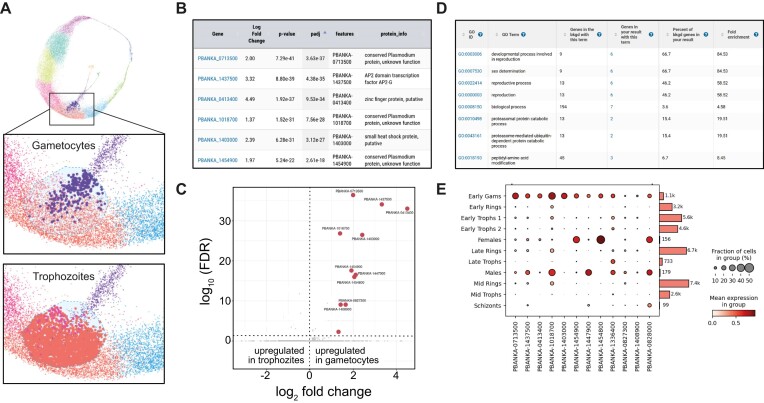
(**A**) The selection of two cell populations (early gametocytes versus early/late trophozoites) in CELLxGENE. (**B**) DEG analysis results table (early gametocytes versus early/late trophozoites). (**C**) Volcano plot of the differentially expressed (DE) genes. (**D**) GO enrichment results table for the DE gene set from PlasmoDB. (**E**) Dot plot showing the relative expression level of each DE gene in each stage of the malaria life cycle.

The utility of paraCell’s interconnectivity is demonstrated here by the re-analysis of scRNA-seq *P. berghei* data produced by Hentzschel *et al.* [9]. The original study charted the sexual development of *Plasmodium* parasites within host cells of varying maturity. Following this, a paraCell atlas linked to the PlasmoDB database [30] was created to present the dataset to the public (https://cellatlas-cxg.mvls.gla.ac.uk/Plasmodium_berghei-Multi.Tissue/) and used to explore genes that are differentially expressed at the early stages of *Plasmodium* gametogenesis (Fig. 1).

In order to assess the robustness of paraCell, we identified 200 genes differentially expressed between reticulocytes and normocytes, replicating an analysis from the original publication (see fig. 5B in [9]). Although small differences were observed between the original results and those produced via paraCell, this can most likely be attributed to the pseudo-bulk strategy used in the original paper. Overall, the results overlap well, and all relevant biological hits were replicated (Supplementary Fig. S1).

Base CELLxGENE functionality was used to target the intersection between the trophozoite stage and the early gametocytes (Fig. 1A). CELLxGENE VIP functionality was then used to perform differential gene expression analysis between trophozoite and early gametocyte cells within this intersection (Fig. 1B).

Several genes known to characterize *Plasmodium* gametogenesis, such as AP2G (PBANKA_1437550), gametocyte development gene 1 (GDV1, PBANKA_0828000), and male development proteins 2 (MD2, PBANKA_1447900) and 3 (MD3, PBANKA_0413400), were found to be upregulated in gametocytes (Fig. 1C). In line with this, exporting the set of DE genes to PlasmoDB via paraCell for GO analysis revealed an enrichment of terms related to sexual determination as well as reproductive and developmental processes (Fig. 1D).

Notably, this analysis also identified several novel genes upregulated in gametocytes but not previously associated with *Plasmodium* gametogenesis, such as PBANKA_0713500, a conserved protein of unknown function, which was the most significant hit of all DEGs, or PBANKA_1403000, a putative small heat shock protein. These genes were found to be primarily expressed in gametocytes, both early in development and in mature male and female cells (Fig. 1E).

These results demonstrate the synergy paraCell promotes between CELLxGENE, CELLxGENE VIP, and external databases (e.g. PlasmoDB). Utilizing the functionality of all three systems enables a highly targeted, interactive analysis, which has identified new candidate genes potentially implicated in the early stages of *Plasmodium* gametogenesis, all without any need for programming experience on the user’s part.

### Case study 3: trajectory inference and trajectory-based differential expression analysis

paraCell expands the functionality of VIP to include both trajectory inference and trajectory-based differential expression analysis. The Trajectory Inference tab can display precomputed Slingshot [31] trajectories, as well as generate PAGA [32] maps describing the overall topology and connectivity of annotated scRNA-seq data. The TradeSeq [26] tab implements the eponymous tool, an R analysis package that enables trajectory-based differential expression analysis. This tab is capable of both displaying pre-computed tradeSeq results and generating new tradeSeq plots indicating the relationship between the expression of a given gene and the progression of a specified lineage.

Together these features allow paraCell atlases to extract and display a wider range of information from accompanying papers and empower users to explore scRNA-seq datasets in new ways.

**Figure 2.**
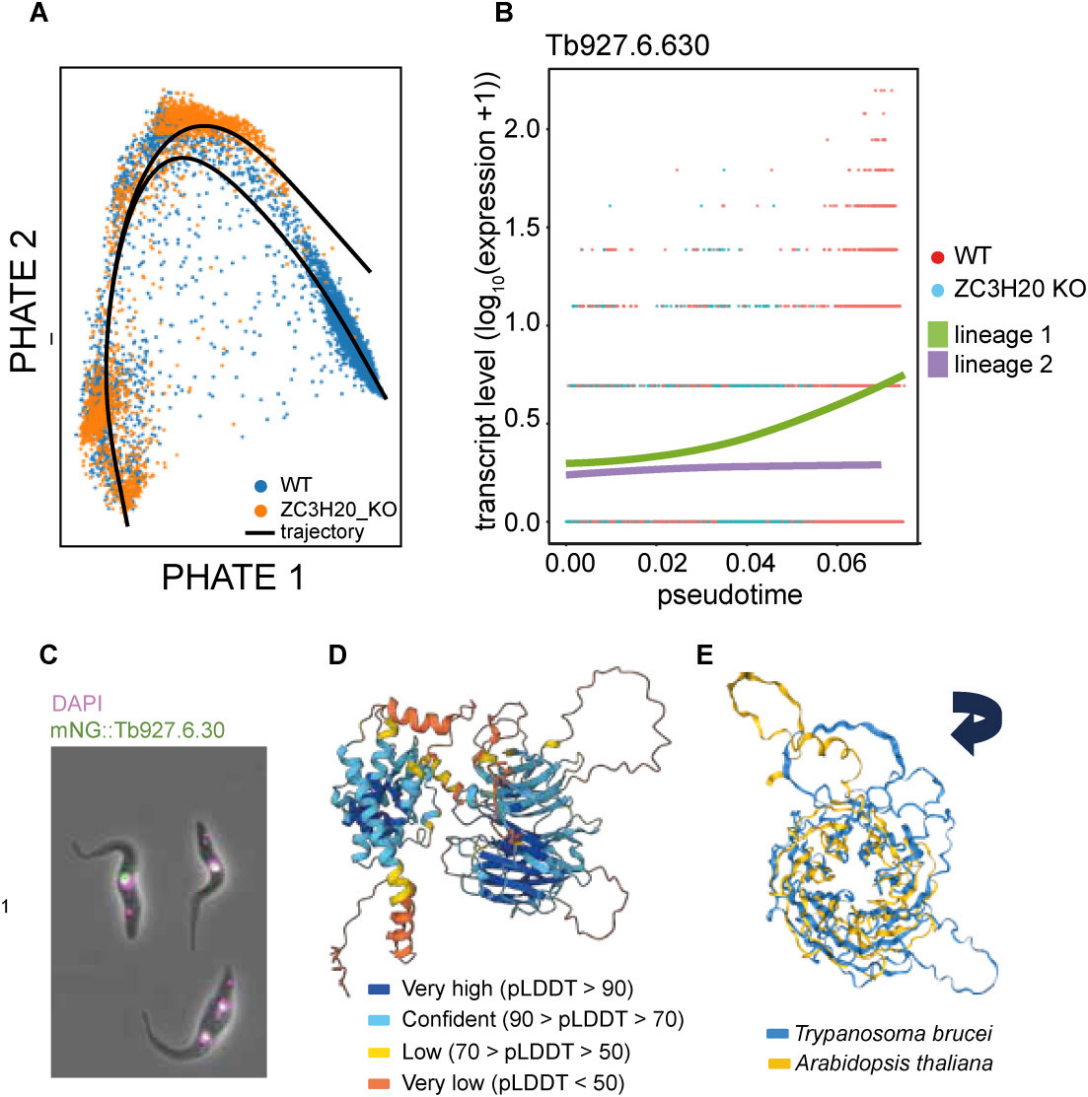
Investigation of a novel life cycle regulated *T. brucei* protein. (**A**) Low-dimensional plot (PHATE) of the *T. brucei*scRNA-seq dataset, grouped by cell type, as presented by the ‘Trajectory Inference’ tab in paraCell. The branched trajectories identified in the data are indicated by black lines. (**B**) mRNA level (*y*-axis) changes for Tb927.6.630 across pseudotime (*x*-axis). mRNA count for individual WT (red) and ZC3H20 null mutant (ZC3H20 KO, teal) *T. brucei* during life cycle progression from slender to stumpy forms. Trajectory 1 of WT parasites (WT 1, green) and alternative trajectory 2 taken by ZC3H20 mutants (ZC3H20 KO 2, purple) show differential expression patterns during full development to stumpy forms in WT parasites and truncated development of mutant parasites. (**C**) Localization of NeonGreen endogenously N-terminal tagged Tb927.6.630 protein (mNG: Tb927.6.630) to the nucleolus of the procyclic form of *T. brucei*. Nuclei visualized by 4',6-diamidino-2-phenylindole (DAPI) staining DNA. Image retrieved from the TrypTag database resource [33]. (**D**) Predicted protein structure, coloured by per-residue confidence score (pLDDT). Image retrieved from the AlphaFold Protein Structure Database [34]. (**E**) Protein alignment of the WD40 repeat-containing domain of *T. brucei* Tb927.6.630 and *Arabidopsis thaliana* protein KTN80.1.

The utility of these features is demonstrated here by the re-analysis of scRNA-seq data generated and published by Briggs *et al.* [8]. The original study described the development of bloodstream form *Trypanosoma brucei* parasites from a replicative ‘slender’ form to a transmissible ‘stumpy’ form in both a WT and a ZC3H20 knockout (KO) population, where the deletion of this critical RNA-binding protein prevents stumpy formation [8]. Two trajectories were identified via Slingshot—a complete trajectory describing the successful differentiation of WT parasites and a truncated trajectory describing the incomplete differentiation of KO parasites (Fig. 2A). Genes significantly associated with each trajectory were then identified via tradeSeq.

Following this, a paraCell atlas was created to present the results to the public (https://cellatlas-cxg.mvls.gla.ac.uk/Trypanosoma_brucei-slender2stumpy/), and the TradeSeq tab was used to identify new genes associated with the successful differentiation of *T. brucei* parasites.

The search for genes differentially expressed across only the WT trajectory from slender to stumpy forms, and not differentially expressed during the KO trajectory, implicated 358 currently uncharacterized genes encoding hypothetical proteins linked to slender to stumpy life cycle development. As noted in the original publication [8], this comparison again identified numerous genes known to be differentially regulated during the slender to stumpy form transition (including EP1 and EP2 procyclin surface protein transcripts, e.g. [35]), as well as ZC3H20 itself, which, as expected, validates our approach. Expression patterns of the hypothetical protein-encoding genes were then plotted using paraCell, allowing further investigation. Here we discuss one gene of interest, Tb927.6.630, revealed by this approach. The tradeSeq plot generated for Tb927.6.630 revealed that the expression of the gene peaks near the endpoint of the WT trajectory, a time point corresponding to the transmissible stumpy form of the parasite (Fig. 2B). Mining other available *T. brucei* resources identified that the predicted protein encoded by Tb927.6.630 is localized to the nucleus of the bloodstream and procyclic form [36], and specifically to the nucleolus of procyclics (Fig. 2C), as revealed by the fluorescent protein tagging project, TrypTag [33]. This protein is predicted to have a complex structure (Fig. 2D) that, when aligned to the database of all available protein structures via FoldSeek [37], returns uncharacterized homolog proteins in related trypanosomatids such as *Trypanosoma cruzi* (Tc00.1047053510535.10) and *Leishmania infantum* (LINF_220022400). A partial alignment was also obtained to the *A. thaliana* protein Katanin p80 WD40 repeat-containing subunit B1 homolog (KTN80.1). It has predicted functions in the rapid reorganization of cellular microtubules [38] (Fig. 2E) and is the non-catalytic subunit of the microtubule-severing enzyme complex katanin [38].

Tb927.6.630 is predicted to contain seven WD40 repeat regions that together form a circularized β-propeller. A similar structure has been documented in KTN80.1 and across the homologous p80 proteins in animal cells, where the WD40 domain targets katanin to the centrosome [39]. Together, this information highlights a complex protein that may have yet unexplored functions, possibly linking structural changes to nuclear microtubules to *T. brucei* life cycle development.

In summary, paraCell has given the ability of all users to easily visualize and analyse the trajectory (using Trajectory Inference and TradeSeq tabs) of this, and other, dataset(s) of interest. We demonstrate how the additional options facilitate the re-analysis of published data and, by doing so, encourage the generation of new biological insights.

### Case study 4: analysing parasite (*Toxoplasma)–*host (mouse) interactions

The Host–Parasite Interactions tab enables the analysis and visualization of dual scRNA-seq host–pathogen datasets. These datasets are essential for exploring intracellular pathogen functionality and are characterized by representing parasite and host gene expression data for each individual cell. Thus, paraCell allows users to subset and visualize the underlying data object, facilitating the comparison of the host and parasite reactive response at the gene expression level. This functionality is demonstrated first with a *Toxoplasma–*mouse atlas and secondly with a *Theileria–*cow atlas.

To study *Toxoplasma–*mouse cell interactions, we extracted and differentiated murine BMDMs, which were unstimulated or stimulated with IFN-γ and infected with type 1, RH *T. gondii*.

After 10× Chromium with Illumina sequencing, we obtained 1600 and 1400 cells for the WT and IFN-γ-stimulated infected cells, respectively. In the WT, we detected 3900 mouse genes and 950 parasite genes per cell. For the IFN-γ-stimulated infected cells, 3100 mouse genes and 850 parasite genes were obtained per cell. Data were processed using Seurat in R (see the ‘Materials and methods’ section), and after filtering, retained 1407 and 1258 cells for the WT and treated, respectively. The Seurat object was converted to an AnnData file and loaded into paraCell (https://cellatlas-cxg.mvls.gla.ac.uk/Toxoplasma_gondii-murine.bone.marrow.derived.macrophages/).

**Figure 3.**
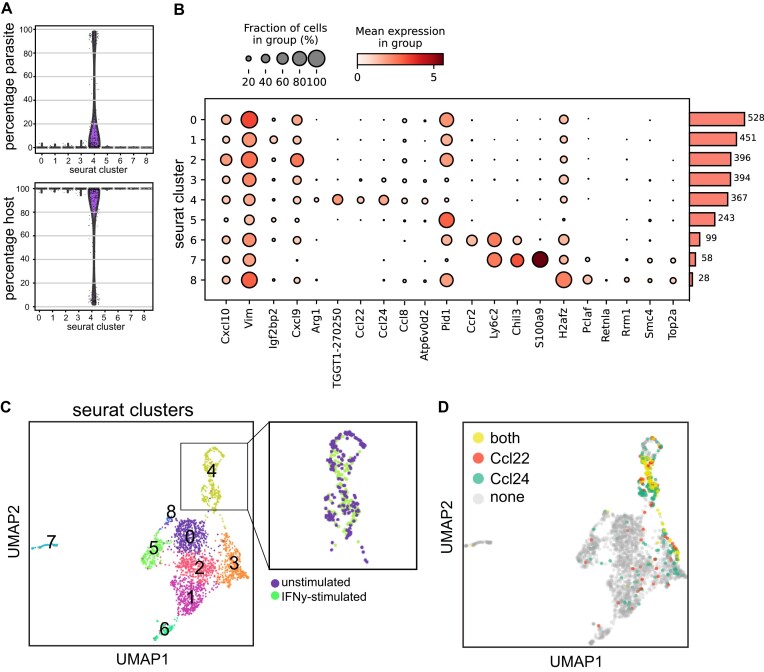
Overview of *Toxoplasma–*mouse cell atlas. (**A**) Violin plot of percentage of parasite cells (above) and host cells (below) in the dataset, by Seurat cluster. (**B**) Dot plot summarizing expression of marker genes for annotation and analysis. The size of the dot reflects how many cells the gene is expressed in for a specific cluster. The colour is the mean expression, and the bar plot at the right indicates the total number of cells per cluster. (**C**) UMAP clustered data in paraCell with Seurat clusters indicated. Inset shows both unstimulated and IFN-γ-stimulated cells in cluster 4 containing infected macrophages. (**D**) Dual gene colouring of UMAP indicating cells with expression of Ccl22, Ccl24, or both.

Often, users receive well-annotated objects to work with. However, here we show that paraCell can be used to identify clusters and to explore how parasites modulate the host cells reactive response. The first analysis was to check the parasite frequency, showing that the cells in most clusters represent primarily host genes, as we only retrieved ∼100 parasite cells per run (Fig. 3A). This was largely due to a low initial multiplicity of infection and the comparatively low level of parasite transcripts, especially in relation to the mouse data for highly active INF-activated macrophages.

The effects of IFN stimulation could clearly be seen in the DEG analysis between runs, with an upregulation of IFN-γ-stimulated (e.g. Irf1, Gbp2, Gbp4, and Stat1) genes in run 2 (IFN) (Supplementary Fig. S2). From the combined UMAP, multiple macrophage populations could be identified based on Seurat clusters (Fig. 3B). Cluster 3 cells expressed a strong signature of IFN-γ stimulation (high levels of Cxcl9, Cxcl10, and Vim). Cluster 6 expressed markers for inflammatory monocytes (Ly6c2 and Ccr2), cluster 7 expressed markers of M2 macrophages (including Chil3 and Retnla), and cluster 8 contained cycling macrophages, expressing multiple genes involved in DNA replication such as Smc4, Top2a, Pclaf, and Rrm1.

Cluster 4, notably, contains *T. gondii* infected macrophages (Fig. 3C), from both stimulated and unstimulated conditions (Fig. 3C, inset). These cells have upregulated Ccl22 and Ccl24 (Fig. 3D), as previously described [40], but also contain a novel marker gene, Atp6v0d2, which is the most highly expressed gene linked to the cluster (Fig. 3B). Interestingly, cluster 4 cells also express Ccl8 (also called MCP-2). Expression of Atp6v0d2 or Clc8 was independent of IFN-γ stimulation but appeared to be linked with parasite infection. Ccl8 is a chemoattractant factor expressed on a range of inflammatory cells and has not previously been associated with *T. gondii* infection. Ccl8 production is, however, known to be upregulated in *Mycobacteria* infection [41] and has recently been shown to be elevated in response to lactate [42]. *Toxoplasma gondii* is known to secrete lactate into the host cell through FNT1 [43], but secretion of lactate [43] was not previously linked to cytokine secretion. Thus, the insights from paraCell allow the identification of a potential important parasite-dependent modulatory event that requires experimental validation. Moreover, the available paraCell functions allowed this analysis to be performed by a first-time user of single-cell data with minimal training, highlighting the usability of the paraCell interface and its ability to allow testable biological insights by end users of the data.

### Case study 5: analysing parasite (*Theileria*)–host (bovine) interactions

As a second host–parasite example, we utilized two *Theileria annulata* infected bovine macrophage cell lines derived from two cow breeds that display differential susceptibility to disease, tropical theileriosis. One cow breed (Holstein) is known to be susceptible to acute pathology upon parasite infection, while the Sahiwal breed is, in general, tolerant. We performed scRNA-seq on the infected cells and obtained around 9000 cells, with ∼4000 genes per host cell and 480 versus 667 genes for the parasite (in Holstein versus Sahiwal, respectively). Data were processed in R using Seurat (as described earlier), leaving us with 8433 and 7831 cells for Holstein and Sahiwal, respectively. In contrast to the *Toxoplasma* example, using paraCell, we could conclude that most of the host cells were infected with parasite (Supplementary Fig. S3, assessed via ‘Host–Parasite Violins’ plotting function). Additionally, host and parasite marker genes can be identified for categorical annotation levels, showing a high percentage of parasites in clusters 4 and 6. These features provide users with an overview of host- and/or parasite-specific gene expression within a dataset.

**Figure 4.**
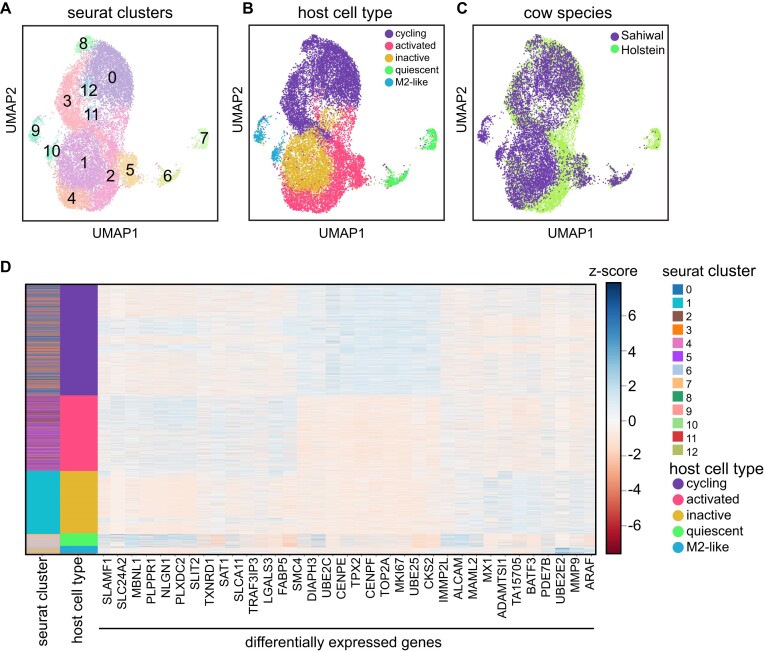
Cow–*Theileria* combined UMAP, produced by reducing dimensions using both host and parasite genes in Seurat, showing clusters (**A**), macrophage subtypes (**B**), and cattle species (**C)**. (**D**) Heatmap showing genes DE for each macrophage subtype.

paraCell can identify different cell clusters (Fig. 4A); based on marker genes (Fig. 4B), some clusters are merged because they exhibit a similar macrophage state signature: clusters 0, 3, 8, and 11 are cycling macrophages expressing the marker MKI67; clusters 9 and 10 are tissue repair M2-like macrophages expressing MMP9/COX2; clusters 2, 4, 5, and 12 are macrophages actively responding to the infection, expressing LGALS3 and TRAF3IP3; and clusters 6 and 7 are non-activated macrophages (Fig. 4D and Supplementary Fig. S4A). The percent expression of host and/or parasite genes within categorical annotations can be assessed via host–parasite violin plots (Supplementary Fig. S3).

**Figure 5.**
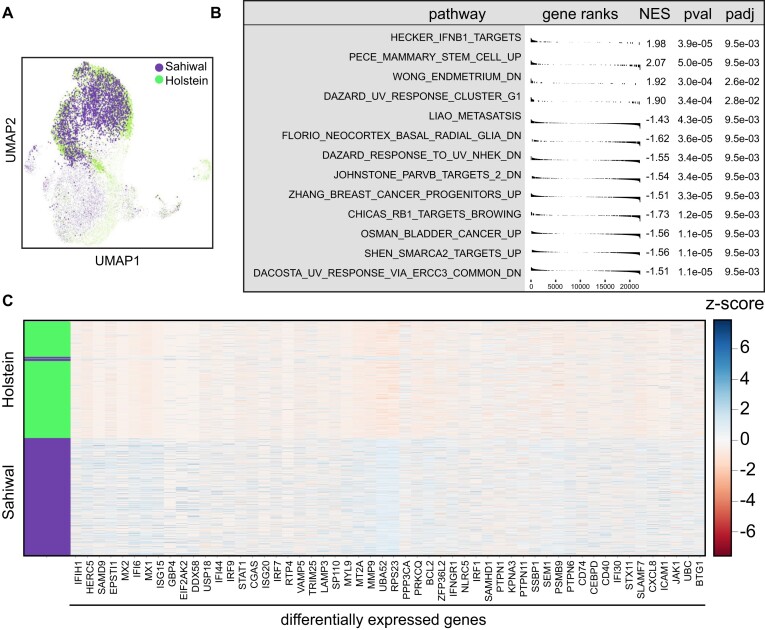
Gene enrichment analysis of cycling macrophages between Sahiwal and Holstein (A) Cow–*Theileria* combined UMAP, showing Sahiwal and Holstein clusters. (**B**) GSEA showing pathway with >0 NES score upregulated in Sahiwal, and <0 upregulated in Holstein. (**C**) Heatmap showing all the genes DE involved in at least one interferon pathway statistically significant for GSEA.

paraCell’s features also enable users to explore the relationship between host and parasite gene expression in an interactive and intuitive manner. In this case study, infected cells from Sahiwal cattle (*Bos indicus*), which are tolerant to the infection, were compared to cells from Holstein cattle (*Bos taurus*), which are susceptible to acute disease. Despite both cell types being infected by *Theileria annulata* and reaching a similar cycling stage (Fig. 4B and C), differential expression analysis between the cycling cells of the two cell lines (Fig. 5A) shows striking differences. The GSEA indicates an active IFN response in the Sahiwal cells during the cell cycling stage, compared to Holstein cells (Fig. 5B). We used paraCell’s option to download tabular data of the GSEA results as CSV, and the results can be found in Supplementary Table S1. The heatmap showing all the DE genes involved in at least one interferon pathway are statistically significant for GSEA clearly shows higher expression levels in Sahiwal compared to Holstein (Fig. 5C). Conversely, the Holstein cells exhibit a gene signature indicative of a pro-carcinogenic profile, with several cancer-associated GO terms, such as ‘SHEN_SMARCA2_TARGETS_UP’ and ‘OSMAN_BLADDER_CANCER_UP’ among the top 10 (Fig. 5B), corroborating previous literature [28]. Specific genes such as USP1, MACF1, HLTF, SMC3, ACTR3, ARHGAP5, CYBB, LGALS3, and TCF7L2 indeed link these GO terms to differentially expressed genes in Holstein, all of which have well-documented roles in cancer (Supplementary Fig. S4B).

Another visualization option of paraCell is the Host–Parasite UMAPs view, which uses interactive Plotly graphs. The box or lasso select function in Plotly allows for freeform selection of cell subsets, dynamically updating graphs. The selections made on the ‘source’ graph (e.g. the Parasite UMAP) automatically update the ‘target’ graph (e.g. the Host UMAP). Selected cell IDs on the source graph are extracted to either highlight the corresponding cells on the target graph or to produce a new version of the target graph displaying only the selected cells, as illustrated in Supplementary Fig. S5.

In summary, paraCell provides a robust and interactive platform for the detailed exploration of HPIs at the single-cell level, offering valuable insights into the gene expression dynamics and reactive responses in infected cellular populations.

## Discussion

In recent years, there has been a push for the adoption of findability, accessibility, interoperability, and reusability (FAIR) data-sharing principles in science [44], which emphasize the utility of automated data retrieval and the importance of making published data easily available for re-use by the wider academic community.

However, the adoption of these principles has been slow in bioinformatics, hindered by the rapid pace of technological development and the frequent need for specialist knowledge during data analysis. Software tools and programming languages are frequently updated, resulting in compatibility issues that can make replicating older analyses on new systems difficult, even when the original analysis code is available. Successfully navigating this complexity can often require significant computational know-how, preventing large segments of the biological community from effectively exploring and/or re-using published datasets. We have addressed this issue in the field of parasitology by implementing paraCell, an interactive cell atlas platform with advanced functionalities and links to external database systems.

paraCell expands the range of experimental results that can be presented by a CELLxGENE atlas to include trajectory inference and tradeSeq, letting scientists easily share more comprehensive versions of their scRNA-seq data. It also integrates CELLxGENE gene sets and incorporates links to a selection of external database systems, streamlining the user experience and enabling cross-system analysis pipelines. Additionally, paraCell adds new analysis and visualization options for both conventional and dual scRNA-seq data, directly increasing the utility of the plugin and addressing the paucity of analysis tools for host–parasite data.

paraCell does all of this within the user-friendly interface provided by CELLxGENE and CELLxGENE VIP and, as such, requires no programming ability to use. This allows it to serve as a bridge between biologists who generate single-cell data and bioinformaticians who often perform the data analysis and annotation, facilitating collaboration between wet and dry lab scientists required for hypothesis-led single-cell transcriptomic studies. We aim to continue implementing methodology to improve paraCell functionality by, for example, using alternative differential expression methods to overcome current limitations of available tests and include novel technological advances in areas such as spatial biology. At the same time, updates on CELLxGENE and CELLxGENE VIP will be included in future versions, expanding the functionality of this tool.

paraCell facilitates the generation of novel insights from previously published data, as illustrated by two of the use cases presented in this report. In the malaria example, paraCell confirmed existing knowledge and enabled the identification of several genes with temporal expression patterns associated with progression towards *Plasmodium* gametogenesis, including a protein of unknown function found to be highly upregulated in early development. In the *T. brucei* example, paraCell enabled the detection of several uncharacterized genes with dynamic transcript level changes during the differentiation of the parasite into its transmissible form. In both cases, paraCell analysis uncovered several candidates that can be further experimentally investigated to uncover if, and how, they are involved in the corresponding biological processes.

We also presented novel host–parasite data and described the first attempts aiming to improve representation of HPI data at the single-cell level. paraCell was used to annotate, analyse, and visualize dual scRNA-seq data and represents an improvement on existing tools (Table 1). For example, in the *Toxoplasma* example, we were able to identify ATP6V_0_d2 as significantly upregulated in infected macrophages. ATP6V_0_d2 is a macrophage-specific subunit of the V-ATPase, and upregulation has previously been identified from bulk RNAseq in *Toxoplasma* (e.g. [45]); however, it has never been highlighted. In *Leishmania*, the gene is essential for vacuole expansion, likely due to its role in cholesterol biosynthesis [46] and may have a related function in the *T. gondii* infected cell [46]. In the *Theileria* example, the first scRNA-seq *Theileria–*cow dataset known to date, we detect relative differences between an activated interferon response versus a pro-carcinogenic phenotype, depending on the genetic background of host breeds linked to disease susceptibility. Although those findings are limited by the lack of replicates, they do highlight the possibility of analysing single-cell datasets to characterize reactive responses of infected cells. They are unique because of their host–parasite nature and should be helpful for the community in generating hypotheses and delineating HPIs that determine the outcome of infection.

## Conclusion

paraCell is a novel extension of CELLxGENE VIP that incorporates multiple new analysis, visualization, and search options, in addition to improving the plugin’s interoperability with both the underlying CELLxGENE functionality and external database systems. Here, we have demonstrated that paraCell enables the generation of new biological insights from published data without requiring any programming ability or highly specialized knowledge on the part of the user, facilitating the re-use of published datasets in future research. Furthermore, the two host–parasite datasets we presented highlight the power of paraCell to identify important reactive responses linked to establishment of the infected cell and disease pathology.

## Supplementary Material

gkaf091_Supplemental_Files

## Data Availability

All the datasets used to demonstrate paraCell functionality can be found as publicly available cell atlases, listed in the following list. For best performance, the Chrome web browser is recommended. The atlas for the case studies are as follows: *Plasmodium berghei*: https://cellatlas-cxg.mvls.gla.ac.uk/Plasmodium_berghei-Multi.Tissue/, from Hentzschel *et al.* [9]. *Trypanosoma brucei*: https://cellatlas-cxg.mvls.gla.ac.uk/Trypanosoma_brucei-slender2stumpy/, from Briggs *et al.* [8]. *Toxoplasma*–Mouse: https://cellatlas-cxg.mvls.gla.ac.uk/Toxoplasma_gondii-murine.bone.marrow.derived.macrophages/ (E-MTAB-14453). *Theileria–*Cow https://cellatlas-cxg.mvls.gla.ac.uk/Theileria_annulata-Bos_indicus_taurus_macrophage/ (E-MTAB-14450). The newly generated raw fastq data are available at ENA: *Toxoplasma–*mouse (E-MTAB-14453) and *Theileria–*cow (E-MTAB-14450).

## Notes

*Code availability*: paraCell is an open-source project and MIT licensed application, available at https://github.com/sii-cell-atlas/paraCell/ and on DOI: 10.5281/zenodo.14758641.

A Docker image of paraCell is also available at https://hub.docker.com/r/edwardagboraw/paracell.

## Notes

*Tutorial*: Comprehensive guidance on the installation and usage of paraCell is available on the paraCell Wiki, available at https://github.com/sii-cell-atlas/paraCell/wiki.
