# Supplementary material for: paraCell: a novel software tool for the interactive analysis and visualization of standard and dual host–parasite single-cell RNA-seq data
Source: Nucleic Acids Res. 2025 Feb 20;53(4):gkaf091. doi: 10.1093/nar/gkaf091 (PMC11840555; doi:10.1093/nar/gkaf091)
Supplement: gkaf091_Supplemental_Files [file gkaf091_supplemental_files.zip › ParaCell.Supplementalmaterial.pdf]

Supplementary material

# paraCell: A novel software tool for the interactive analysis and visualisation of standard and dual host-parasite single-cell RNA-seq data

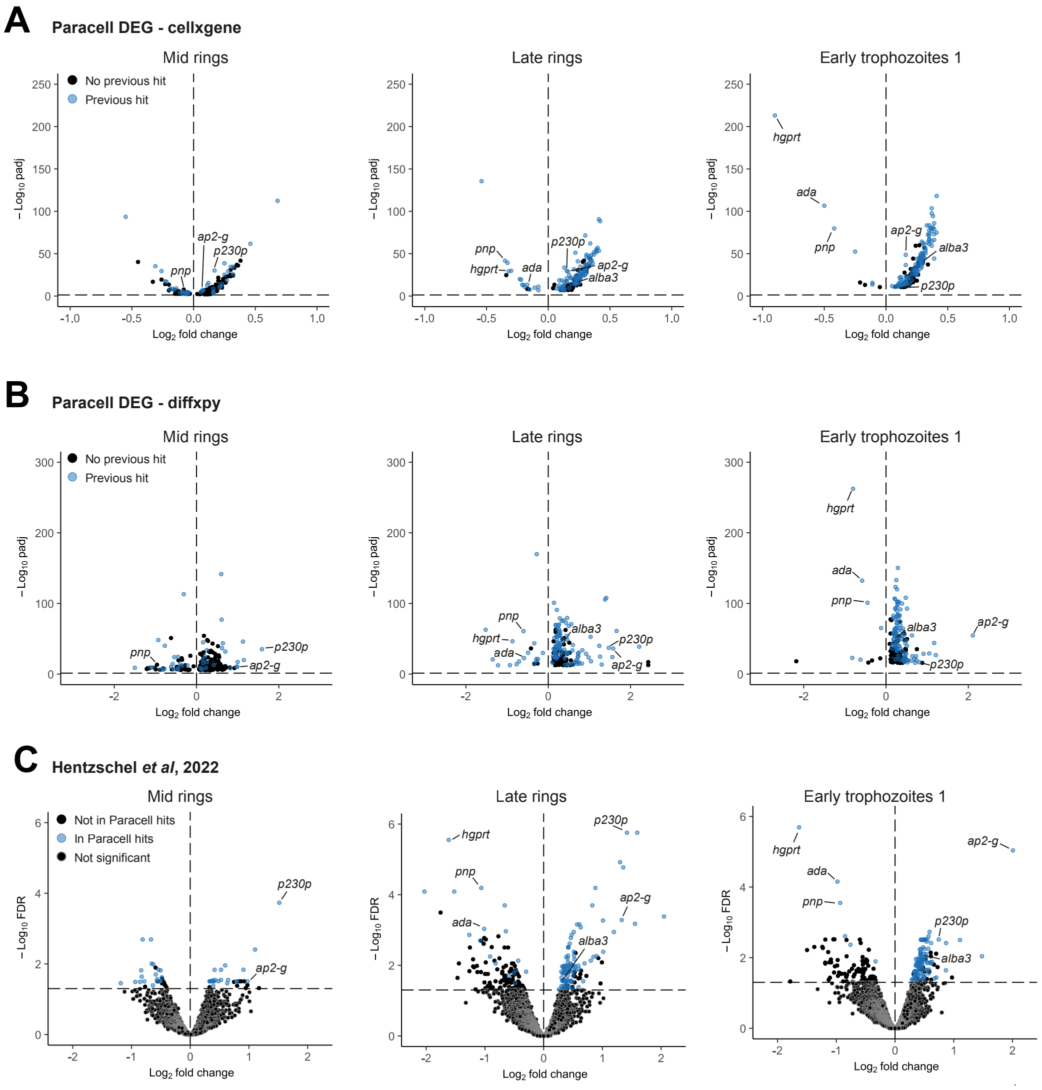


**Supplementary Figure S1:** Comparison of DEG identified with paraCell with hits from previous work (Hentzschel *et al*., 2022 (8)) of reticulocytes versus normocytes in three malaria life stages. A) and B) compare paraCell over the top 200 genes found with the CELLxGENE and diffxpy platforms, for three life cycle stages. C) compares with hits denoted in the original work.

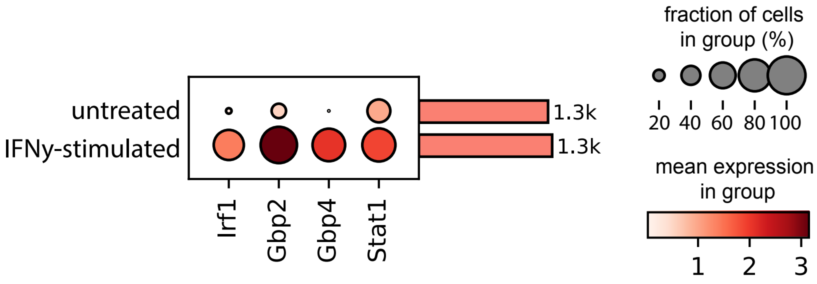


**Supplementary Figure S2**: Expression dot-plots for the *Toxoplasma*-mouse atlas. Genes upregulated due to IFN stimulation

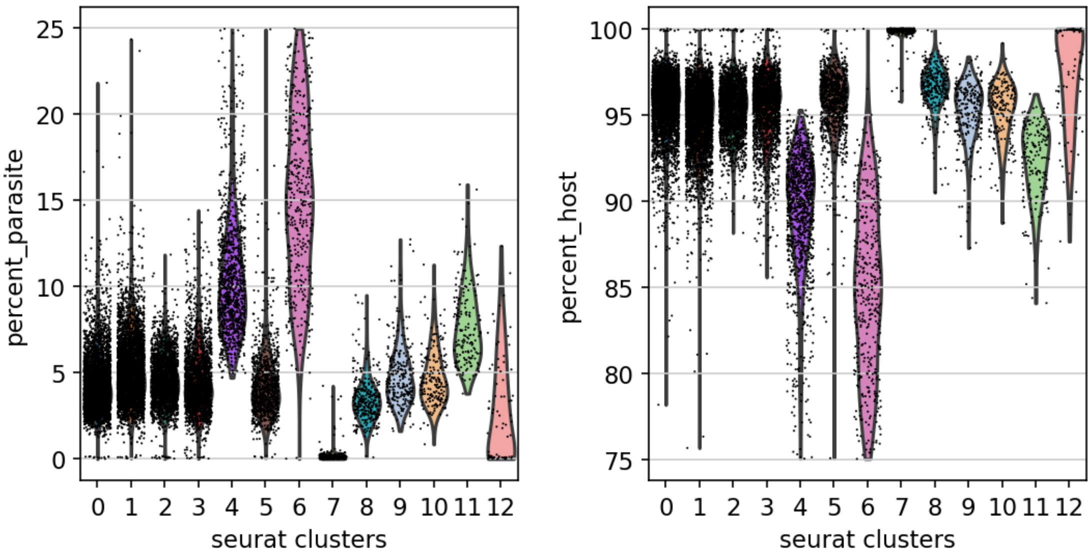


**Supplementary Figure S3:** Violin plots showing the frequency of mRNA in *Theileria* (left panel) versus cow (right panel) for the different Seurat (cell) clusters.

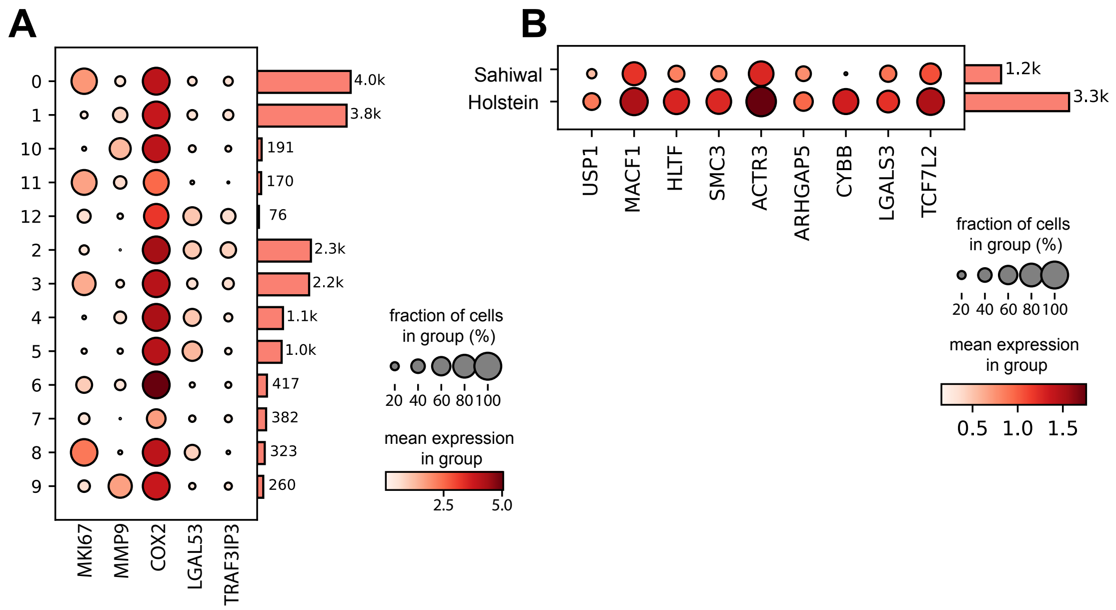


**Supplementary Figure S4**: Gene expression, based on mean expression values, in the annotated *Theileria*-cow atlas. A) Marker genes utilised for the annotation of the host cluster. B) Genes associated with cancer are highly expressed in Holstein.

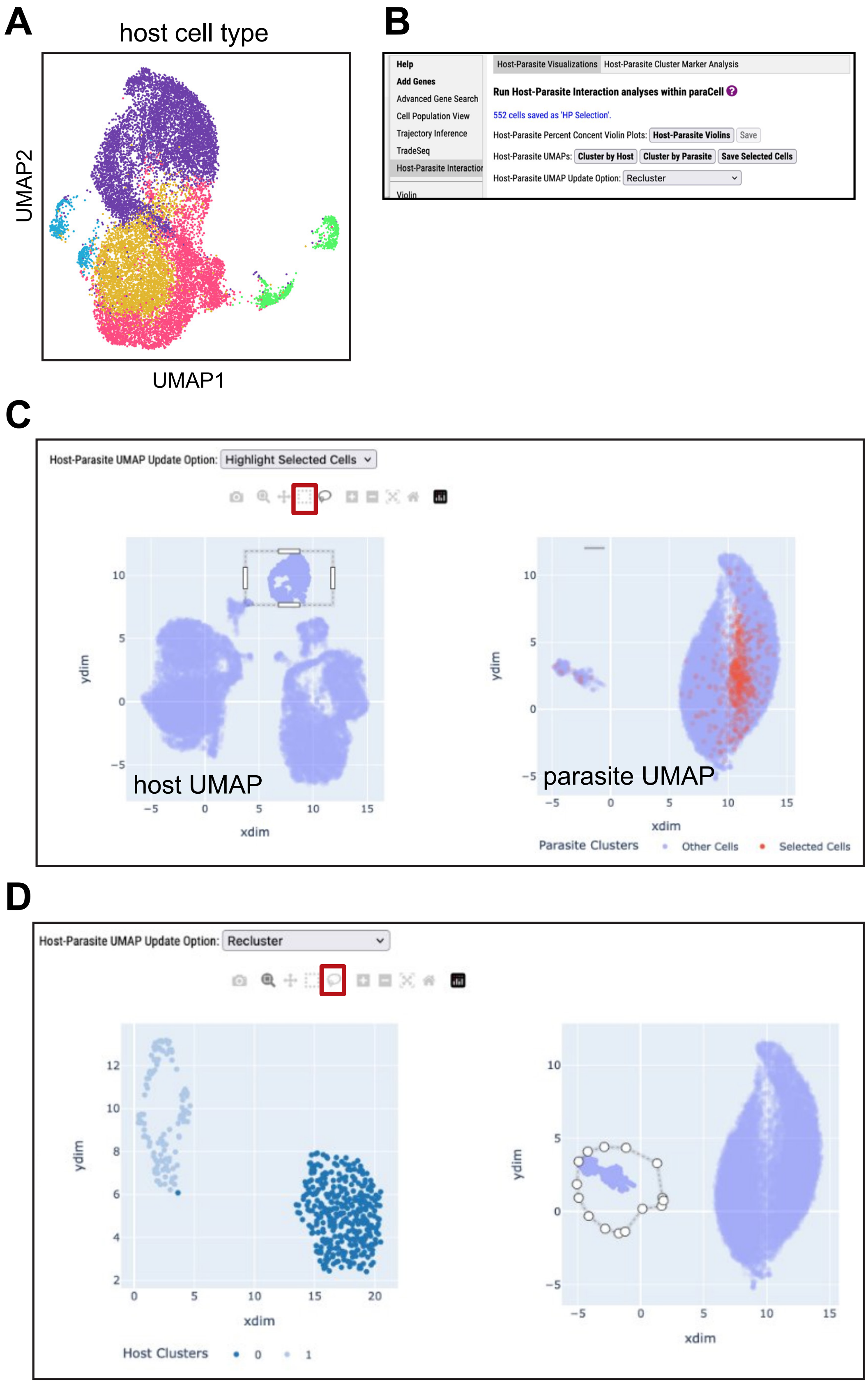


**Supplementary Figure S5**: Analysis performed through the Host-Parasite Interaction (HPI) tab. It allows the user to split the host and parasite data. A) UMAP was used for the analysis.

Every point represents a host cell, which contains a parasite. B) The view of the HPI tab. The user needs first to select the cluster by host and then the cluster by parasite. C) In the Host UMAP, each point represents a host cell without the parasite. Right are the parasite cells without the host. If the user selects the “Highlight Selected Cells” option and selects cells in the Host UMAP with the box function, the parasite cells that they “contain” in (A) are highlighted in red in the parasite UMAP. The user can now click the “Save Selected Cells” button (B) and the cells will be saved as “HP Selection” D) Use of the “Recluster” option: In the parasite UMAP cells are selected via the lasso function. Then, the Host UMAP gets updated, re-clustering the Host cells in which the parasites are contained.
